# Supplementary material for: Reduced Activity of Soluble Fibroblast Activation Protein (sFAP) Represents a Biomarker of Aggressive Disease in Lymphoid Malignancies
Source: Int J Mol Sci. 2025 Nov 21;26(23):11248. doi: 10.3390/ijms262311248 (PMC12692083; doi:10.3390/ijms262311248)
Supplement: Supplementary file 1 [file ijms-26-11248-s001.zip › ijms-3873056-supplementary.pdf]

Supplementary material

Table S1. *Clinicopathological features of the patient subgroup with available tissue biopsies for immunohistochemical evaluation*

Characteristics	DLBCL n=23 n (%)	FL n=11 n (%)
Sex		
Male	14 (61)	7 (64)
Female	9 (39)	4 (36)
Age at diagnosis, y		
Mean	70	65
Range	20-91	33-85
Comorbidities		
No	4 (17)	3 (27)
Yes	18 (78)	8 (73)
Unknown	1 (4)	0 (0)
Ann Arbor Stage		
I-II	6 (26)	3 (27)
III-IV	17 (74)	8 /73)
Unknown/Not relevant	0 (0)	0 (0)
B-Symptoms		
No	11 (48)	9 (82)
Yes	12 (52)	2 (18)
ECOG		
< 2	13 (57)	11 (100)
≥ 2	10 (43)	0 (0)
LDH-elevation		
No	14 (61)	10 (91)
Yes	9 (39)	1 (9)
Nodal sites		
< 2	5 (22)	7 (64)
≥ 2	18 (78)	4 (36)
Bulky disease		
No	18 (78)	9 (82)
Yes	5 (22)	2 (18)
BM involvement		
No	21 (91)	7 (64)
Yes	2 (9)	3 (27)
Unknown	0 (0)	1 (9)
IPI		
0-I	4 (17)	3 (27)
II-III	7 (30)	6 (55)
IV-V	12 (52)	0 (0)
Unknown/Not relevant	0 (0)	2 (18)

BM, bone marrow; DLBCL, diffuse large B-cell lymphoma; FL, follicular lymphoma; IPI, international prognostic index; LDH, lactate dehydrogenase; y, years.

Supplementary Figure S1

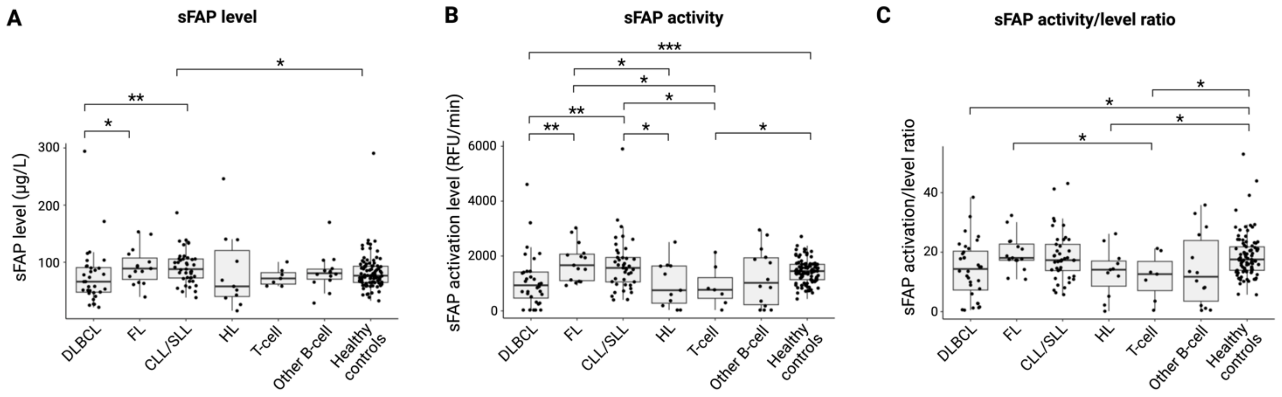

Figure S1: sFAP levels, sFAP activity, and ratio corrected sFAP between entity subgroups and healthy individuals. Presentation of the individual malignant entities and healthy individuals and their serum **A)** sFAP levels (µg/L), **B)** sFAP activity (RFU/min), and **C)** corrected sFAP activity/sFAP level ratio. *p<0.05; **p<0.01; ***p<0.001. Abbreviations: CLL/SLL, chronic lymphocytic leukemia/small lymphocytic lymphoma; DLBCL, diffuse large B-cell lymphoma; FL, follicular lymphoma; HL, Hodgkin's lymphoma; RFU, relative fluorescent units; sFAP, soluble fibroblast activation protein; TL/L, T-cell lymphoma/leukemia.

Supplementary Figure S2

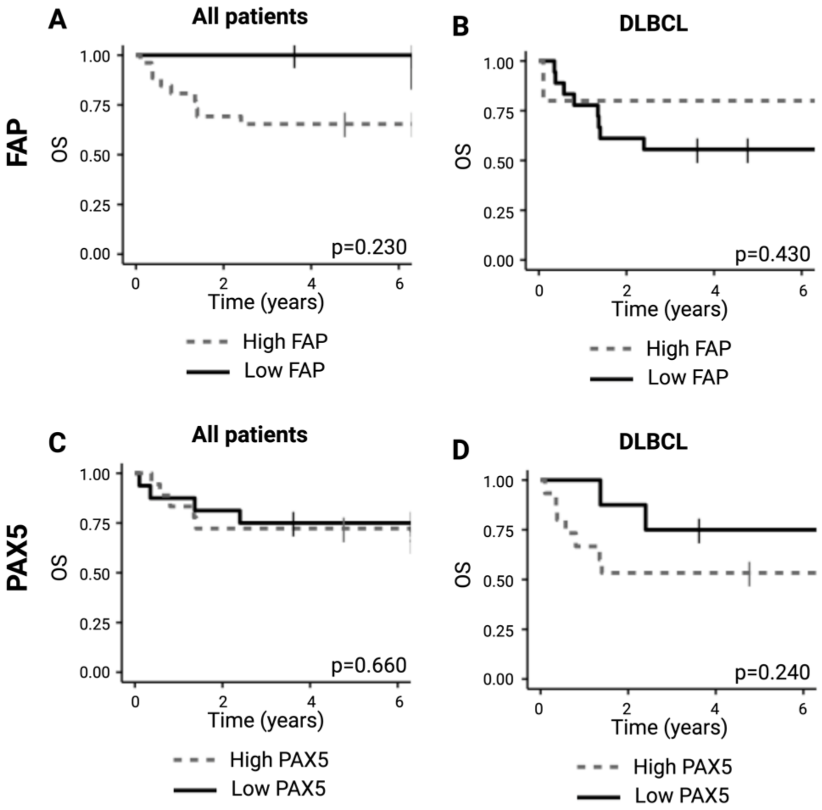

Figure S2. *Outcome analysis according to tumor tissue expression of FAP and PAX5, respectively, in DLBCL and FL* ***A*)** Analysis of overall survival (OS) according to FAP expression in the complete study group of DLBCLs and FLs (cutoff AF=0.0142). **B)** Analysis of OS according to FAP expression in the subgroup of DLBCL (cutoff AF=0.5087). **C)** Analysis of OS according to PAX5 expression in the complete study group of DLBCLs and FLs (cutoff AF=0.1731). **D)** Analysis of OS according to PAX5 expression in the subgroup of DLBCL (cutoff AF=0.2875). Abbreviations: DLBCL, diffuse large B-cell lymphoma; FAP, fibroblast activation protein; FL, follicular lymphoma; OS, overall survival; PAX5, paired box 5 protein.
